# Lost in translation—what is translational neuroscience research?

**DOI:** 10.1093/braincomms/fcag229

**Published:** 2026-07-01

**Authors:** Laurent Sheybani, David Belin

**Affiliations:** Geneva, Switzerland; Cambridge, UK

## Abstract

Our editors discuss and define translational neuroscience research, a concept central to the identity of *Brain Communications.*

*Brain Communications* was launched in 2019 under the leadership of Professor Tara Spires-Jones, a world expert in the cellular and molecular bases of neurodegenerative disorders, especially Alzheimer’s disease. The ambition was to become home to robust translational neuroscience research. Professor David Belin, a behavioural neuroscientist focusing on the biobehavioural basis of the individual vulnerability to develop impulsive/compulsive spectrum disorders such as addiction or obsessive/compulsive disorder, was recently appointed as the new editor-in-chief of *Brain Communications*. With this recent appointment, we, i.e. Laurent Sheybani, an expert in epilepsy and sleep science and long-standing associate editor of the journal, and David Belin, thought it timely to open a discussion about how we think about translational neuroscience.

Many views have been expressed on what constitutes translational neuroscience research, suggesting some confusion about this concept, which lies at the heart of *Brain Communications*’ identity. While discussing the matter with colleagues, certain questions keep coming up. Should translational neuroscience necessarily refer to causal, mechanistic approaches to understand brain disease? Or can one assume that observational neuroscience can also be considered translational? Others have questioned whether a study that includes only humans, or only non-human animals, can be considered translational or whether a study without obvious and/or immediate clinical relevance can be claimed to be translational.

The concept of translational research seems to have gained momentum in the early 1990s to fill a gap in scientific funding.^1^ To promote research that can help develop new therapeutic tools for the treatment of human diseases, scientists in the field of oncology used this term to bring candidate drugs from bench to bedside—and to secure the financial resources to do so.^1^ This was a clever ‘marketing’ strategy for something genuinely useful, while also serving as a pragmatic approach for academic institutions and funding agencies to steer clinical research.^1^ Since then, the translational nature of research in biological and biomedical sciences has been a key feature of scientific publications and grant applications.

The translational nature of research is strongly promoted by funding agencies, which consider it part of the quantitative impact assessment of the research programmes they support. Any relatively superficial survey of funding calls and universities’ programmes will highlight how much *translational* research is being encouraged. However, the same quick overview also reveals that while most definitions refer to research findings that can be *translated* into new treatments,^2,3^ some subtleties exist. For example, some include the development of technologies that can be utilized in medicine within the scope of translational research.^4^ Others encourage funding of start-ups as an objective of translational grants.^5,6^ Others are rather focused on ‘basic, disease-related studies’.^7^ Do these views systematically apply to neuroscience? Is there a single framework that encompasses translational research in neuroscience?

The liberty with which the term translational neuroscience is used in academia and beyond warrants a clear and exhaustive definition of the concept, which is neither trivial nor a vain intellectual exercise.^8^ One potential consensus definition of translational neuroscience, then, is the one offered by Horn *et al*.^9^: ‘a systematic, theory-driven approach that aims to develop and leverage basic and clinical neuroscientific knowledge to aid the development and optimization of clinical and public health interventions’.^9^ The core principle of translational neuroscience research is therefore to produce knowledge that is directly transferable from bench to bedside—and, increasingly, back again. This field is expected to yield new insights into the biological basis of neurological and psychiatric disorders, thereby enabling the development of novel, more effective preventive and therapeutic strategies and informing public health policies.

A study may be inherently translational, but in many cases it may not, and its translational significance may emerge only from its combination with other, sometimes yet-to-be-published, studies. We are committed to ensuring that *Brain Communications* offers the most fertile ground for the papers it harbours to contribute eventually to this emergent property of translational neuroscience research. To that aim, we intend to bring together, under the same conceptual umbrella, the four pillars of translational neuroscience research: (i) basic neuroscience (cells, circuits, molecules, computation), (ii) preclinical models and human experimental paradigms, (iii) clinical research (patients, biomarkers, clinical trials) and (iv) implementation (healthcare systems, policy, public health).

There may not be a better illustration of this emergent property than the case of the discovery of hypothalamic orexin neurons, the loss of which is central to the pathogenesis of narcolepsy, a pathology characterized notably by sudden sleep attacks.^10-12^ The first evidence for the existence of that neuronal population was reported in a 1996 paper published in *PNAS.*^13^ In this paper, the primary goal was to map protein content of the hypothalamus using cDNA library screening. After the initial discovery of a clone, so-called ‘clone 35’, that was exclusively expressed in the hypothalamus, this clone was identified as hypocretin,^14^ also called orexin, because of its role in the regulation of feeding behaviour.^15^ Fourteen years later, in 2022, a study demonstrated the therapeutic potential of orexin receptor agonists for the treatment of narcolepsy^16^ and a phase 3 trial reported improved sleep in people with insomnia following administration of an orexin receptor antagonist.^17^ While the clinical potential of the first discovery was far from obvious, it led, through the accretion of a wealth of fundamental and then translational papers, to life-changing treatments less than two decades later.

We hope that *Brain Communications* will provide fertile ground for such success stories by promoting active discussions about the translational relevance of data published in our journal. Thus, because we believe in the emergent nature of translational neuroscience, we do not expect our colleagues who submit their neuroscience work to *Brain Communications* to make any explicit claim about its translational nature. Instead, we hope that our community will come together to reveal the translational potential of everything we publish.

To summarize, at *Brain Communications*, we welcome all neuroscience research, be it fundamental, behavioural, clinical, *in vivo, in vitro, in silico* or indeed translational… and we, as a community, including through letters to the editors, scientific commentaries, and any other communication platform available, will help the magic happen: uncover their contribution to advancing translational neuroscience.

The cover image for this issue shows noradrenergic locus coeruleus neurons (red) which express GCaMP (green) selectively in cells which project to the nucleus accumbens. Image courtesy of Chloe Chernoff and David Belin.
